# Bis(6-meth­oxy-2-{[tris­(hydroxy­meth­yl)methyl-κ*O*]imino­meth­yl}phenolato-κ^2^ *N*,*O* ^1^)nickel(II) dihydrate

**DOI:** 10.1107/S1600536809022028

**Published:** 2009-06-17

**Authors:** Tian Zhou, Ru-Jin Zhou, Zhe An

**Affiliations:** a School of Chemistry and Life Science, Maoming University, Maoming 525000, People’s Republic of China

## Abstract

In the title compound, [Ni(C_12_H_16_NO_5_)_2_]·2H_2_O, the Ni^II^ atom is coordinated by four O atoms and two N atoms from the two 6-meth­oxy-2-{[tris­(hydroxy­meth­yl)meth­yl]imino­meth­yl}phenolate ligands in a distorted octa­hedral coordination geometry. O—H⋯O hydrogen bonds link the complexes and uncoordinated water mol­ecules into two-dimensional networks parallel to (001).

## Related literature

For the applications of Schiff-base complexes, see: Kritagawa & Kondo (1998 ▶); Zhang *et al.* (1998 ▶); Yaghi *et al.* (1996 ▶). 
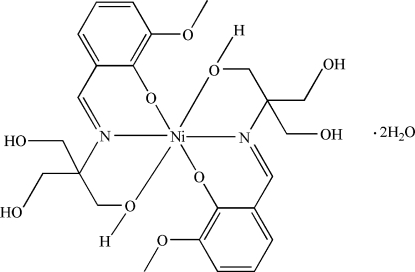


## Experimental

### 

#### Crystal data

[Ni(C_12_H_16_NO_5_)_2_]·2H_2_O
*M* *_r_* = 603.26
Monoclinic, 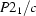

*a* = 12.0142 (10) Å
*b* = 10.9876 (10) Å
*c* = 20.324 (2) Å
β = 97.501 (1)°
*V* = 2660.0 (4) Å^3^
*Z* = 4
Mo *K*α radiation
μ = 0.80 mm^−1^
*T* = 293 K
0.44 × 0.29 × 0.20 mm

#### Data collection

Bruker APEXII CCD diffractometer
Absorption correction: multi-scan (*SADABS*; Sheldrick, 2003 ▶) *T*_min_ = 0.721, *T*_max_ = 0.857
13321 measured reflections
4933 independent reflections
4436 reflections with *I* > 2σ(*I*)
*R*_int_ = 0.043

#### Refinement

*R*[*F* ^2^ > 2σ(*F* ^2^)] = 0.039
*wR*(*F* ^2^) = 0.117
*S* = 1.00
4933 reflections
376 parameters
8 restraints
H atoms treated by a mixture of independent and constrained refinement
Δρ_max_ = 0.38 e Å^−3^
Δρ_min_ = −0.44 e Å^−3^

### 

Data collection: *APEX2* (Bruker, 2004 ▶); cell refinement: *SAINT-Plus* (Bruker, 2001 ▶); data reduction: *SAINT-Plus*; program(s) used to solve structure: *SHELXS97* (Sheldrick, 2008 ▶); program(s) used to refine structure: *SHELXL97* (Sheldrick, 2008 ▶); molecular graphics: *SHELXTL* (Sheldrick, 2008 ▶); software used to prepare material for publication: *SHELXTL*.

## Supplementary Material

Crystal structure: contains datablocks I, global. DOI: 10.1107/S1600536809022028/bi2378sup1.cif

Structure factors: contains datablocks I. DOI: 10.1107/S1600536809022028/bi2378Isup2.hkl

Additional supplementary materials:  crystallographic information; 3D view; checkCIF report

## Figures and Tables

**Table 1:** Hydrogen-bond geometry (Å, °)

*D*—H⋯*A*	*D*—H	H⋯*A*	*D*⋯*A*	*D*—H⋯*A*
O1—H1⋯O2^i^	0.82	1.85	2.670 (3)	179
O2—H2*A*⋯O11^ii^	0.82	1.91	2.666 (3)	152
O2—H2*A*⋯O12^ii^	0.82	2.37	3.010 (3)	135
O5—H5⋯O6^iii^	0.82	1.87	2.691 (3)	174
O6—H6⋯O3^iv^	0.82	1.89	2.671 (2)	159
O10—H10*A*⋯O5^iv^	0.82 (3)	1.93 (3)	2.751 (3)	175 (5)
O8—H1*AA*⋯O7^i^	0.82 (2)	1.972 (11)	2.775 (4)	166 (4)
O4—H4*AA*⋯O8^v^	0.82 (3)	1.88 (4)	2.686 (3)	170 (4)
O8—H1*BB*⋯O2^vi^	0.82 (3)	2.16 (3)	2.962 (3)	167 (4)
O7—H2*BB*⋯O9^ii^	0.82 (2)	2.055 (10)	2.862 (4)	168 (4)
O7—H2*AA*⋯O1	0.81 (3)	1.84 (3)	2.641 (3)	169 (4)

Symmetry codes: (i) 
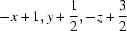
; (ii) 
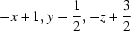
; (iii) 
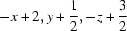
; (iv) 
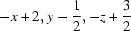
; (v) 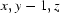; (vi) 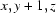.

**Fig. 1.**
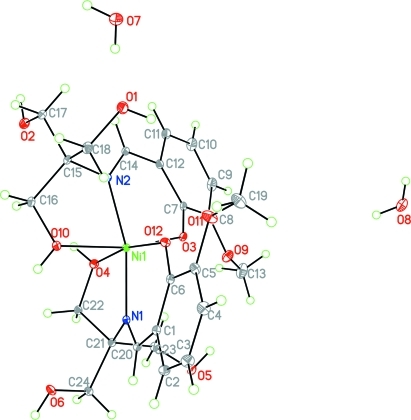
Molecular structure with displacement ellipsoids drawn at the 30% probability level for non-H atoms.

**Fig. 2.**
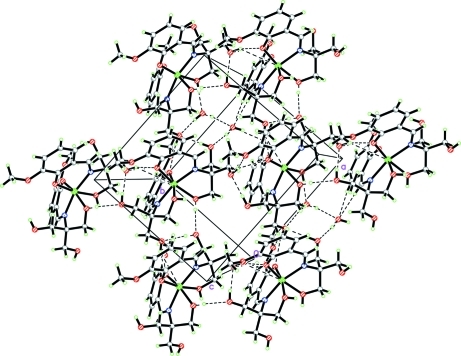
Packing diagram viewed approximately along the c axis, showing the complex network of O—H···O hydrogen bonds (dashed lines).

## supplementary crystallographic information

### Comment

Polymeric metal complexes containing Schiff-base ligands are of interest because of their useful chemical or physical properties (Zhang *et al.*, 1998; Kritagawa & Kondo, 1998; Yaghi *et al.*, 1996). Herein, we report a new crystal structure containing the Schiff-base ligand 6-methoxy-2-{[tris(hydroxymethyl)methyl]iminomethyl}phenol (denoted H*L*).

As shown in Figure 1, the asymmetric unit of the complex comprises two *L*^-^ ligands, one Ni^II^ atom and two lattice water molecules. The Ni^II^ atom is hexa-coordinated by four O atoms and two N atoms from the two *L*^-^ ligands, giving a distorted octahedral coordination geometry. The Ni—O and Ni—N bond distances are within normal ranges. The [Ni*L*_2_] complexes form an extensive network of O—H···O interactions involving the lattice water molecules, giving 2-D networks parallel to the (001) planes (Fig. 2).

### Experimental

The complex was synthesized by refluxing H*L* (0.050 g, 0.2 mmol) and NiCl_2_.6H_2_O (0.048 g, 0.2 mmol) in the mixed solution (CH_3_OH:H_2_O = 4:1) until all solid was dissolved. The solution was then cooled to room temperature and filtered. Green crystals for X-ray diffraction analysis were obtained by slow evaporation of the filtrate. Elemental analysis calculated: C 47.74, H 5.97, N 4.64 %; found: C 47.69, H 5.51, N 4.58 %.

### Refinement

All H atoms bound to C were placed geometrically with C—H = 0.93 (aromatic H), 0.96 (methyl H) or 0.97 Å (methylene H) and refined as riding with *U*_iso_(H) = 1.2*U*_eq_(C) (aromatic and methylene H) or 1.5*U*_eq_(C) (methyl H). The H atoms of the water molecule were located from difference density maps and refined with distance restraints of d(H···H) = 1.38 (2) Å, d(O—H) = 0.82 (1) Å. The H atoms of the hydroxyl groups were placed geometrically with O—H = 0.82 Å.

### Crystal data

**Table:**

[Ni(C_12_H_16_NO_5_)_2_]·2H_2_O	*F*(000) = 1272
*M**_r_* = 603.26	*D*_x_ = 1.506 Mg m^−^^3^
Monoclinic, *P*2_1_/*c*	Mo *K*α radiation, λ = 0.71073 Å
Hall symbol: -P 2ybc	Cell parameters from 4933 reflections
*a* = 12.0142 (10) Å	θ = 2.0–25.5°
*b* = 10.9876 (10) Å	µ = 0.80 mm^−^^1^
*c* = 20.324 (2) Å	*T* = 293 K
β = 97.501 (1)°	Block, green
*V* = 2660.0 (4) Å^3^	0.44 × 0.29 × 0.20 mm
*Z* = 4	

### Data collection

**Table:**

Bruker APEXII CCD diffractometer	4933 independent reflections
Radiation source: fine-focus sealed tube	4436 reflections with *I* > 2σ(*I*)
graphite	*R*_int_ = 0.043
φ and ω scans	θ_max_ = 25.5°, θ_min_ = 2.0°
Absorption correction: multi-scan (*SADABS*; Sheldrick, 2003)	*h* = −14→11
*T*_min_ = 0.721, *T*_max_ = 0.857	*k* = −13→13
13321 measured reflections	*l* = −24→21

### Refinement

**Table:**

Refinement on *F*^2^	Primary atom site location: structure-invariant direct methods
Least-squares matrix: full	Secondary atom site location: difference Fourier map
*R*[*F*^2^ > 2σ(*F*^2^)] = 0.039	Hydrogen site location: inferred from neighbouring sites
*wR*(*F*^2^) = 0.117	H atoms treated by a mixture of independent and constrained refinement
*S* = 1.00	*w* = 1/[σ^2^(*F*_o_^2^) + (0.069*P*)^2^ + 2.387*P*] where *P* = (*F*_o_^2^ + 2*F*_c_^2^)/3
4933 reflections	(Δ/σ)_max_ = 0.032
376 parameters	Δρ_max_ = 0.38 e Å^−^^3^
8 restraints	Δρ_min_ = −0.44 e Å^−^^3^

### Special details

**Table:**

Geometry. All e.s.d.'s (except the e.s.d. in the dihedral angle between two l.s. planes) are estimated using the full covariance matrix. The cell e.s.d.'s are taken into account individually in the estimation of e.s.d.'s in distances, angles and torsion angles; correlations between e.s.d.'s in cell parameters are only used when they are defined by crystal symmetry. An approximate (isotropic) treatment of cell e.s.d.'s is used for estimating e.s.d.'s involving l.s. planes.
Refinement. Refinement of *F*^2^ against ALL reflections. The weighted *R*-factor *wR* and goodness of fit *S* are based on *F*^2^, conventional *R*-factors *R* are based on *F*, with *F* set to zero for negative *F*^2^. The threshold expression of *F*^2^ > σ(*F*^2^) is used only for calculating *R*-factors(gt) *etc*. and is not relevant to the choice of reflections for refinement. *R*-factors based on *F*^2^ are statistically about twice as large as those based on *F*, and *R*- factors based on ALL data will be even larger.

### Fractional atomic coordinates and isotropic or equivalent isotropic displacement parameters (Å^2^)

**Table:**

	*x*	*y*	*z*	*U*_iso_*/*U*_eq_	
C1	0.9480 (2)	0.2452 (2)	0.86933 (11)	0.0233 (5)	
C2	1.0283 (2)	0.2677 (3)	0.92480 (12)	0.0311 (6)	
H2	1.1023	0.2433	0.9242	0.037*	
C3	0.9989 (3)	0.3243 (3)	0.97874 (13)	0.0386 (7)	
H3	1.0524	0.3390	1.0153	0.046*	
C4	0.8871 (3)	0.3610 (3)	0.97952 (14)	0.0391 (7)	
H4	0.8671	0.4003	1.0168	0.047*	
C5	0.8073 (2)	0.3402 (2)	0.92655 (13)	0.0307 (6)	
C6	0.8335 (2)	0.2797 (2)	0.86879 (11)	0.0228 (5)	
C7	0.6684 (2)	0.3019 (2)	0.62935 (12)	0.0231 (5)	
C8	0.6897 (3)	0.3685 (2)	0.57276 (13)	0.0325 (6)	
C9	0.6097 (3)	0.3783 (3)	0.51866 (15)	0.0468 (8)	
H9	0.6251	0.4238	0.4823	0.056*	
C10	0.5051 (3)	0.3210 (3)	0.51678 (16)	0.0515 (9)	
H10	0.4509	0.3307	0.4801	0.062*	
C11	0.4834 (3)	0.2522 (3)	0.56833 (15)	0.0404 (7)	
H11	0.4143	0.2136	0.5668	0.048*	
C12	0.5639 (2)	0.2379 (2)	0.62428 (12)	0.0269 (5)	
C13	0.8357 (4)	0.4634 (4)	0.52069 (18)	0.0638 (11)	
H13A	0.8300	0.3993	0.4883	0.096*	
H13B	0.9128	0.4872	0.5312	0.096*	
H13C	0.7921	0.5319	0.5030	0.096*	
C14	0.5356 (2)	0.1521 (2)	0.67266 (13)	0.0265 (5)	
H14	0.4621	0.1238	0.6679	0.032*	
C15	0.5631 (2)	0.0166 (2)	0.76457 (13)	0.0264 (5)	
C16	0.6607 (2)	−0.0731 (2)	0.77949 (14)	0.0312 (6)	
H16A	0.6454	−0.1291	0.8141	0.037*	
H16B	0.6694	−0.1199	0.7400	0.037*	
C17	0.4562 (2)	−0.0513 (3)	0.73736 (15)	0.0344 (6)	
H17A	0.4381	−0.1104	0.7698	0.041*	
H17B	0.3943	0.0059	0.7296	0.041*	
C18	0.5411 (3)	0.0770 (3)	0.82811 (14)	0.0370 (6)	
H18A	0.5226	0.0156	0.8592	0.044*	
H18B	0.6083	0.1190	0.8478	0.044*	
C19	0.6632 (3)	0.4399 (3)	0.97696 (15)	0.0488 (8)	
H19A	0.6755	0.3908	1.0163	0.073*	
H19B	0.5850	0.4603	0.9678	0.073*	
H19C	0.7068	0.5131	0.9835	0.073*	
C20	0.9938 (2)	0.1930 (2)	0.81394 (12)	0.0228 (5)	
H20	1.0712	0.1823	0.8182	0.027*	
C21	0.9989 (2)	0.1161 (2)	0.70684 (12)	0.0229 (5)	
C22	0.9245 (2)	0.0216 (2)	0.66780 (12)	0.0260 (5)	
H22A	0.9502	0.0080	0.6251	0.031*	
H22B	0.9290	−0.0549	0.6918	0.031*	
C23	1.0163 (2)	0.2240 (2)	0.66147 (12)	0.0276 (5)	
H23A	1.0622	0.1977	0.6282	0.033*	
H23B	0.9441	0.2490	0.6386	0.033*	
C24	1.1143 (2)	0.0572 (2)	0.72948 (13)	0.0282 (5)	
H24A	1.1428	0.0193	0.6919	0.034*	
H24B	1.1675	0.1189	0.7475	0.034*	
N1	0.93758 (17)	0.16035 (17)	0.75960 (9)	0.0199 (4)	
N2	0.60221 (17)	0.11097 (18)	0.72161 (10)	0.0228 (4)	
Ni1	0.76754 (2)	0.15968 (3)	0.742167 (14)	0.01963 (12)	
O1	0.4508 (2)	0.1618 (2)	0.81592 (14)	0.0531 (6)	
H1	0.4759	0.2313	0.8178	0.080*	
O2	0.46970 (17)	−0.11101 (19)	0.67775 (11)	0.0418 (5)	
H2A	0.4079	−0.1255	0.6571	0.063*	
O3	0.74281 (14)	0.30277 (15)	0.68125 (8)	0.0233 (4)	
O4	0.81045 (15)	0.06375 (17)	0.65815 (9)	0.0301 (4)	
O5	1.06823 (16)	0.32512 (17)	0.69579 (10)	0.0341 (4)	
H5	1.0200	0.3734	0.7042	0.051*	
O6	1.10157 (16)	−0.03050 (19)	0.77806 (11)	0.0399 (5)	
H6	1.1596	−0.0702	0.7859	0.060*	
O7	0.2701 (2)	0.1557 (2)	0.87725 (16)	0.0584 (7)	
O8	0.6646 (2)	0.8964 (2)	0.60240 (12)	0.0524 (6)	
O9	0.79479 (19)	0.4219 (2)	0.57859 (10)	0.0440 (5)	
O10	0.76155 (15)	−0.00657 (17)	0.80057 (10)	0.0311 (4)	
O11	0.69602 (18)	0.3743 (2)	0.92284 (10)	0.0446 (5)	
O12	0.75320 (14)	0.26451 (16)	0.82089 (8)	0.0258 (4)	
H10A	0.815 (2)	−0.054 (3)	0.803 (2)	0.080*	
H1AA	0.690 (3)	0.8269 (14)	0.603 (2)	0.080*	
H2AA	0.329 (2)	0.151 (3)	0.862 (2)	0.080*	
H4AA	0.768 (3)	0.008 (3)	0.645 (2)	0.080*	
H1BB	0.605 (2)	0.902 (3)	0.618 (2)	0.080*	
H2BB	0.243 (3)	0.0900 (16)	0.886 (2)	0.080*	

### Atomic displacement parameters (Å^2^)

**Table:**

	*U*^11^	*U*^22^	*U*^33^	*U*^12^	*U*^13^	*U*^23^
C1	0.0281 (12)	0.0218 (12)	0.0198 (11)	−0.0011 (10)	0.0026 (9)	−0.0003 (9)
C2	0.0308 (13)	0.0363 (15)	0.0247 (13)	0.0002 (12)	−0.0020 (10)	0.0005 (11)
C3	0.0432 (17)	0.0480 (18)	0.0220 (13)	−0.0017 (14)	−0.0059 (12)	−0.0053 (12)
C4	0.0487 (18)	0.0460 (17)	0.0222 (13)	0.0024 (14)	0.0036 (12)	−0.0104 (12)
C5	0.0367 (15)	0.0310 (14)	0.0247 (13)	0.0056 (11)	0.0052 (11)	−0.0026 (10)
C6	0.0300 (13)	0.0203 (11)	0.0177 (11)	−0.0016 (10)	0.0022 (9)	0.0016 (9)
C7	0.0293 (13)	0.0164 (11)	0.0232 (12)	0.0003 (10)	0.0022 (10)	−0.0012 (9)
C8	0.0461 (16)	0.0242 (13)	0.0265 (13)	−0.0038 (12)	0.0026 (11)	0.0015 (10)
C9	0.073 (2)	0.0381 (16)	0.0257 (14)	−0.0042 (16)	−0.0061 (14)	0.0096 (12)
C10	0.065 (2)	0.0432 (18)	0.0374 (17)	−0.0052 (16)	−0.0254 (16)	0.0094 (14)
C11	0.0407 (16)	0.0311 (15)	0.0442 (17)	−0.0031 (12)	−0.0144 (13)	0.0020 (12)
C12	0.0294 (13)	0.0217 (12)	0.0276 (12)	0.0018 (10)	−0.0035 (10)	−0.0005 (10)
C13	0.094 (3)	0.053 (2)	0.052 (2)	−0.018 (2)	0.040 (2)	−0.0003 (17)
C14	0.0231 (12)	0.0206 (12)	0.0346 (14)	−0.0017 (10)	−0.0015 (10)	−0.0026 (10)
C15	0.0262 (12)	0.0206 (12)	0.0333 (13)	−0.0046 (10)	0.0068 (10)	0.0036 (10)
C16	0.0318 (14)	0.0220 (13)	0.0391 (14)	−0.0031 (11)	0.0022 (11)	0.0055 (11)
C17	0.0268 (13)	0.0267 (14)	0.0494 (17)	−0.0059 (11)	0.0037 (12)	0.0054 (12)
C18	0.0429 (16)	0.0336 (15)	0.0375 (15)	−0.0038 (13)	0.0170 (12)	0.0044 (12)
C19	0.056 (2)	0.057 (2)	0.0362 (16)	0.0188 (16)	0.0167 (14)	−0.0120 (15)
C20	0.0223 (12)	0.0202 (11)	0.0253 (12)	−0.0011 (10)	0.0011 (9)	0.0006 (9)
C21	0.0248 (12)	0.0220 (12)	0.0225 (11)	0.0003 (10)	0.0056 (9)	−0.0032 (9)
C22	0.0307 (13)	0.0203 (12)	0.0265 (12)	0.0007 (10)	0.0022 (10)	−0.0055 (10)
C23	0.0331 (13)	0.0269 (13)	0.0239 (12)	−0.0017 (11)	0.0080 (10)	−0.0004 (10)
C24	0.0267 (13)	0.0261 (13)	0.0322 (13)	0.0031 (10)	0.0054 (10)	−0.0007 (10)
N1	0.0236 (10)	0.0174 (10)	0.0190 (10)	0.0009 (8)	0.0044 (8)	0.0006 (7)
N2	0.0221 (10)	0.0186 (10)	0.0276 (11)	−0.0009 (8)	0.0028 (8)	−0.0005 (8)
Ni1	0.02003 (18)	0.01864 (18)	0.01981 (18)	−0.00071 (11)	0.00109 (12)	−0.00067 (11)
O1	0.0484 (14)	0.0359 (12)	0.0817 (18)	0.0007 (10)	0.0338 (13)	−0.0071 (12)
O2	0.0364 (11)	0.0328 (11)	0.0528 (13)	−0.0080 (9)	−0.0074 (9)	−0.0070 (10)
O3	0.0269 (9)	0.0198 (8)	0.0223 (8)	−0.0042 (7)	−0.0004 (7)	0.0010 (7)
O4	0.0286 (9)	0.0285 (10)	0.0319 (10)	−0.0018 (8)	−0.0014 (7)	−0.0101 (8)
O5	0.0351 (11)	0.0263 (10)	0.0421 (11)	−0.0068 (8)	0.0096 (9)	−0.0016 (8)
O6	0.0309 (10)	0.0326 (11)	0.0560 (13)	0.0111 (9)	0.0046 (9)	0.0139 (9)
O7	0.0449 (14)	0.0510 (15)	0.0836 (19)	0.0014 (11)	0.0248 (13)	−0.0003 (13)
O8	0.0615 (16)	0.0469 (14)	0.0485 (13)	−0.0191 (12)	0.0057 (11)	−0.0043 (11)
O9	0.0523 (13)	0.0463 (12)	0.0351 (11)	−0.0156 (10)	0.0125 (9)	0.0085 (9)
O10	0.0281 (9)	0.0250 (9)	0.0390 (10)	0.0007 (8)	−0.0004 (8)	0.0041 (8)
O11	0.0417 (12)	0.0646 (14)	0.0275 (10)	0.0182 (11)	0.0046 (9)	−0.0162 (10)
O12	0.0246 (9)	0.0298 (9)	0.0222 (8)	0.0023 (7)	0.0008 (7)	−0.0059 (7)

### Geometric parameters (Å, °)

**Table:**

C1—C2	1.406 (3)	C17—H17B	0.970
C1—C6	1.426 (4)	C18—O1	1.427 (4)
C1—C20	1.435 (3)	C18—H18A	0.970
C2—C3	1.347 (4)	C18—H18B	0.970
C2—H2	0.930	C19—O11	1.414 (3)
C3—C4	1.405 (5)	C19—H19A	0.960
C3—H3	0.930	C19—H19B	0.960
C4—C5	1.364 (4)	C19—H19C	0.960
C4—H4	0.930	C20—N1	1.269 (3)
C5—O11	1.381 (3)	C20—H20	0.930
C5—C6	1.419 (3)	C21—N1	1.461 (3)
C6—O12	1.289 (3)	C21—C22	1.524 (3)
C7—O3	1.290 (3)	C21—C23	1.533 (3)
C7—C8	1.414 (4)	C21—C24	1.545 (3)
C7—C12	1.431 (4)	C22—O4	1.435 (3)
C8—C9	1.366 (4)	C22—H22A	0.970
C8—O9	1.384 (4)	C22—H22B	0.970
C9—C10	1.402 (5)	C23—O5	1.413 (3)
C9—H9	0.930	C23—H23A	0.970
C10—C11	1.345 (5)	C23—H23B	0.970
C10—H10	0.930	C24—O6	1.402 (3)
C11—C12	1.402 (4)	C24—H24A	0.970
C11—H11	0.930	C24—H24B	0.970
C12—C14	1.435 (4)	N1—Ni1	2.027 (2)
C13—O9	1.409 (4)	N2—Ni1	2.047 (2)
C13—H13A	0.960	Ni1—O12	1.9971 (17)
C13—H13B	0.960	Ni1—O3	1.9993 (17)
C13—H13C	0.960	Ni1—O4	2.1266 (18)
C14—N2	1.275 (3)	Ni1—O10	2.1847 (19)
C14—H14	0.930	O1—H1	0.820
C15—N2	1.471 (3)	O2—H2A	0.820
C15—C18	1.506 (4)	O4—H4AA	0.82 (3)
C15—C17	1.526 (3)	O5—H5	0.820
C15—C16	1.531 (4)	O6—H6	0.820
C16—O10	1.432 (3)	O7—H2AA	0.81 (3)
C16—H16A	0.970	O7—H2BB	0.82 (2)
C16—H16B	0.970	O8—H1AA	0.82 (2)
C17—O2	1.406 (4)	O8—H1BB	0.82 (3)
C17—H17A	0.970	O10—H10A	0.82 (3)
			
C2—C1—C6	121.3 (2)	O11—C19—H19B	109.5
C2—C1—C20	114.0 (2)	H19A—C19—H19B	109.5
C6—C1—C20	124.6 (2)	O11—C19—H19C	109.5
C3—C2—C1	120.6 (3)	H19A—C19—H19C	109.5
C3—C2—H2	119.7	H19B—C19—H19C	109.5
C1—C2—H2	119.7	N1—C20—C1	125.5 (2)
C2—C3—C4	119.6 (3)	N1—C20—H20	117.2
C2—C3—H3	120.2	C1—C20—H20	117.2
C4—C3—H3	120.2	N1—C21—C22	106.9 (2)
C5—C4—C3	121.2 (3)	N1—C21—C23	107.81 (19)
C5—C4—H4	119.4	C22—C21—C23	109.3 (2)
C3—C4—H4	119.4	N1—C21—C24	116.1 (2)
C4—C5—O11	125.0 (2)	C22—C21—C24	108.2 (2)
C4—C5—C6	121.5 (3)	C23—C21—C24	108.4 (2)
O11—C5—C6	113.5 (2)	O4—C22—C21	109.65 (19)
O12—C6—C5	117.4 (2)	O4—C22—H22A	109.7
O12—C6—C1	126.7 (2)	C21—C22—H22A	109.7
C5—C6—C1	115.9 (2)	O4—C22—H22B	109.7
O3—C7—C8	118.8 (2)	C21—C22—H22B	109.7
O3—C7—C12	124.8 (2)	H22A—C22—H22B	108.2
C8—C7—C12	116.4 (2)	O5—C23—C21	113.4 (2)
C9—C8—O9	125.0 (3)	O5—C23—H23A	108.9
C9—C8—C7	120.8 (3)	C21—C23—H23A	108.9
O9—C8—C7	114.2 (2)	O5—C23—H23B	108.9
C8—C9—C10	121.3 (3)	C21—C23—H23B	108.9
C8—C9—H9	119.3	H23A—C23—H23B	107.7
C10—C9—H9	119.3	O6—C24—C21	108.7 (2)
C11—C10—C9	119.7 (3)	O6—C24—H24A	109.9
C11—C10—H10	120.2	C21—C24—H24A	109.9
C9—C10—H10	120.2	O6—C24—H24B	109.9
C10—C11—C12	120.8 (3)	C21—C24—H24B	109.9
C10—C11—H11	119.6	H24A—C24—H24B	108.3
C12—C11—H11	119.6	C20—N1—C21	118.1 (2)
C11—C12—C7	120.6 (2)	C20—N1—Ni1	124.24 (17)
C11—C12—C14	115.5 (2)	C21—N1—Ni1	117.62 (15)
C7—C12—C14	123.9 (2)	C14—N2—C15	119.8 (2)
O9—C13—H13A	109.5	C14—N2—Ni1	124.04 (18)
O9—C13—H13B	109.5	C15—N2—Ni1	116.08 (15)
H13A—C13—H13B	109.5	O12—Ni1—O3	91.21 (7)
O9—C13—H13C	109.5	O12—Ni1—N1	92.77 (7)
H13A—C13—H13C	109.5	O3—Ni1—N1	99.80 (7)
H13B—C13—H13C	109.5	O12—Ni1—N2	97.55 (8)
N2—C14—C12	126.0 (2)	O3—Ni1—N2	91.00 (7)
N2—C14—H14	117.0	N1—Ni1—N2	164.91 (8)
C12—C14—H14	117.0	O12—Ni1—O4	169.89 (7)
N2—C15—C18	107.7 (2)	O3—Ni1—O4	85.67 (7)
N2—C15—C17	116.6 (2)	N1—Ni1—O4	78.33 (7)
C18—C15—C17	107.0 (2)	N2—Ni1—O4	92.12 (8)
N2—C15—C16	106.0 (2)	O12—Ni1—O10	91.98 (7)
C18—C15—C16	109.2 (2)	O3—Ni1—O10	168.87 (7)
C17—C15—C16	110.1 (2)	N1—Ni1—O10	90.69 (7)
O10—C16—C15	109.1 (2)	N2—Ni1—O10	78.01 (7)
O10—C16—H16A	109.9	O4—Ni1—O10	92.89 (7)
C15—C16—H16A	109.9	C18—O1—H1	109.5
O10—C16—H16B	109.9	C17—O2—H2A	109.5
C15—C16—H16B	109.9	C7—O3—Ni1	122.09 (15)
H16A—C16—H16B	108.3	C22—O4—Ni1	112.16 (13)
O2—C17—C15	110.7 (2)	C22—O4—H4AA	110 (3)
O2—C17—H17A	109.5	Ni1—O4—H4AA	115 (3)
C15—C17—H17A	109.5	C23—O5—H5	109.5
O2—C17—H17B	109.5	C24—O6—H6	109.5
C15—C17—H17B	109.5	H2AA—O7—H2BB	115 (3)
H17A—C17—H17B	108.1	H1AA—O8—H1BB	114 (3)
O1—C18—C15	110.7 (2)	C8—O9—C13	118.7 (3)
O1—C18—H18A	109.5	C16—O10—Ni1	110.53 (14)
C15—C18—H18A	109.5	C16—O10—H10A	109 (3)
O1—C18—H18B	109.5	Ni1—O10—H10A	118 (3)
C15—C18—H18B	109.5	C5—O11—C19	117.5 (2)
H18A—C18—H18B	108.1	C6—O12—Ni1	123.07 (16)
O11—C19—H19A	109.5		

### Hydrogen-bond geometry (Å, °)

**Table:**

*D*—H···*A*	*D*—H	H···*A*	*D*···*A*	*D*—H···*A*
O1—H1···O2^i^	0.82	1.85	2.670 (3)	179
O2—H2A···O11^ii^	0.82	1.91	2.666 (3)	152
O2—H2A···O12^ii^	0.82	2.37	3.010 (3)	135
O5—H5···O6^iii^	0.82	1.87	2.691 (3)	174
O6—H6···O3^iv^	0.82	1.89	2.671 (2)	159
O10—H10A···O5^iv^	0.82 (3)	1.93 (3)	2.751 (3)	175 (5)
O8—H1AA···O7^i^	0.82 (2)	1.97 (1)	2.775 (4)	166 (4)
O4—H4AA···O8^v^	0.82 (3)	1.88 (4)	2.686 (3)	170 (4)
O8—H1BB···O2^vi^	0.82 (3)	2.16 (3)	2.962 (3)	167 (4)
O7—H2BB···O9^ii^	0.82 (2)	2.06 (1)	2.862 (4)	168 (4)
O7—H2AA···O1	0.81 (3)	1.84 (3)	2.641 (3)	169 (4)

Symmetry codes: (i) −*x*+1, *y*+1/2, −*z*+3/2; (ii) −*x*+1, *y*−1/2, −*z*+3/2; (iii) −*x*+2, *y*+1/2, −*z*+3/2; (iv) −*x*+2, *y*−1/2, −*z*+3/2; (v) *x*, *y*−1, *z*; (vi) *x*, *y*+1, *z*.
